# What’s in a name field?

**DOI:** 10.23889/ijpds.v9i5.2693

**Published:** 2024-09-18

**Authors:** Frances McCarty, Ben Rogers, Jessie Parker, Cordell Golden

**Affiliations:** 1 CDC/NCHS

The National Center for Health Statistics (NCHS) has a data linkage program that combines national survey data with key sources of health outcomes and health care utilization. The overall accuracy and quality of a data linkage depends on the quality of the data fields. This applies in a variety of data linkage methods, including clear text and privacy preserving record linkage. Data pre-processing and cleaning are essential to address data quality issues in most linkage tasks. Automating data cleaning prevents time-consuming manual reviews particularly when linkages involve a large number of records. For some data fields, these activities are relatively straightforward. For example, dates typically have a limited number of plausible values that make checking and cleaning relatively easy and straightforward. Unique identifiers (e.g., social security number) often conform to some set format or have restrictions on the values that would be expected. Other data fields such as first name and last name present greater challenges with respect to automating the cleaning process. For some data sources, first name and last name fields may contain non-name text that needs to be identified and removed. In this project, we examined the use of an artificial intelligence (AI) based large language model (LLM) and a simple rule-based algorithm to identify non-name text in name fields that should be removed prior to linkage. We also investigated the impact of automated name clean-up algorithms on the quality of an example linkage.

